# From tradition to healing: the promise of acupuncture in managing chronic fatigue syndrome

**DOI:** 10.3389/fmed.2025.1724290

**Published:** 2026-01-20

**Authors:** Delong Wang, Tiansong Yang, Yang Cui, Yuanyuan Qu, Chuwen Feng, Zhongren Sun, Miao Zhang

**Affiliations:** 1 Key Laboratory of Neurobiology of Clinical Acupuncture, The Second Affiliated Hospital of Heilongjiang University of Chinese Medicine, Harbin, Heilongjiang, China; 2 Department of Rehabilitation II, The First Affiliated Hospital of Heilongjiang University of Chinese Medicine, Harbin, Heilongjiang, China; 3 Department of Acupuncture VII, The Second Affiliated Hospital of Heilongjiang University of Chinese Medicine, Harbin, Heilongjiang, China; 4 Heilongjiang University of Chinese Medicine, Harbin, Heilongjiang, China; 5 The Second Affiliated Hospital of Heilongjiang University of Chinese Medicine, Harbin, Heilongjiang, China

**Keywords:** acupuncture and moxibustion treatment, CFS, curative effect evaluation, individualized treatment, ME/CFS, pathophysiological mechanism

## Abstract

Chronic fatigue syndrome (CFS) is a global public health problem affecting more than 65 million patients worldwide. The combined prevalence rate of CFS was 45.2% after 4 weeks in patients with novel coronavirus. Women, people over 40 years of age, and low-income people are susceptible groups, which have a significant impact on immune, nervous, endocrine, and other system functions. First, from the perspective of epidemiology, this paper reviews the global epidemic trend of CFS, the differences in incidence and prevalence in different regions and populations, and risk factors such as heredity, infection, and childhood trauma. Second, the development of diagnostic techniques for CFS, including the evolution of clinical diagnostic criteria, research progress on immune and metabolic biomarkers, and the application of MRI and other imaging techniques in the diagnosis of CFS, is described, followed by an in-depth discussion of the genetics of CFS, including genetic susceptibility, genomic association, and familial aggregation. The pathophysiological mechanism of CFS was also analyzed, revealing abnormalities in NK cell function and immune factors in the immune system, dysfunction of the hypothalamic-pituitary-adrenal axis in the neuroendocrine system, and disorders of energy and lipid metabolism in the metabolic system. This paper focuses on the study of acupuncture and moxibustion treatment of CFS, traces back to the historical application of acupuncture and moxibustion treatment of CFS, analyzes the relationship between the pathological mechanism of CFS and acupuncture and moxibustion intervention, expounds the theoretical basis of traditional Chinese medicine and modern mechanism of action of acupuncture and moxibustion treatment, and introduces the results of clinical trials, efficacy evaluation methods, and individualized treatment strategies for acupuncture and moxibustion treatment of CFS. The innovative application of acupuncture techniques, such as electroacupuncture and acupoint catgut embedding, as well as the synergistic effect of acupuncture combined with traditional Chinese medicine and psychotherapy, are shown. At the same time, disputes and challenges in the efficacy, safety, and ethics of acupuncture treatment for CFS were pointed out, and future research directions, potential breakthroughs, and international cooperation opportunities of acupuncture treatment for CFS are discussed. This study provides a comprehensive reference for clinical treatment and research on CFS.

## Introduction

Chronic fatigue syndrome (CFS) is a global public health problem affecting more than 65 million patients worldwide. The combined prevalence of CFS after four weeks in patients with COVID-19 has been reported as 45.2%, with higher rates observed in women, people over 40 years of age, individuals with low income, and other susceptible groups. CFS exerts a significant impact on the immune system, nervous system, and other multisystem functions, leading to substantial impairment in daily life and long-term health.

In recent years, increasing attention has been paid to the epidemiological characteristics and biological underpinnings of CFS. Existing studies have described global epidemic trends, differences in incidence and prevalence across regions and populations, and a range of potential risk factors. Parallel advances in diagnostic methods have focused on the evolution of clinical diagnostic criteria, the development and validation of candidate biomarkers, and imaging diagnostic findings. Genetic research has further explored genetic susceptibility, genomic associations, and familial aggregation patterns, while pathophysiological studies have highlighted abnormal manifestations of the immune, neuroendocrine, and metabolic systems.

Acupuncture and moxibustion, as important components of traditional Chinese medicine, have a long history in the management of CFS and are considered to exert certain therapeutic effects. Clinical practice and research have gradually accumulated evidence on their potential to regulate multiple systems and improve fatigue-related symptoms. In this context, it is necessary to systematically summarize the historical application of acupuncture and moxibustion in CFS, clarify their theoretical basis and current application status, and review available clinical trial data, efficacy evaluation methods, and individualized treatment strategies. At the same time, emerging work on innovative acupuncture and moxibustion techniques, the development of related equipment and tools, and their combination with other treatment modalities warrant integrated discussion.

Based on these, the present review uses a multi-dimensional perspective to summarize and analyze CFS and the role of acupuncture and moxibustion in its treatment by (1) presenting the epidemiological basis of CFS, including global epidemic trends, incidence and prevalence differences, and risk factors; (2) elaborating current diagnostic techniques, encompassing the evolution of clinical diagnostic criteria, progress in biomarker research, and imaging diagnostic findings; (3) describing advances in genetic research, including genetic susceptibility, genomic associations, and familial aggregation; (4) analyzing the pathophysiological mechanisms of CFS to reveal abnormal manifestations of the immune, neuroendocrine, and metabolic systems; and (5) tracing the historical application of acupuncture and moxibustion in CFS, explaining their theoretical basis and application status, introducing clinical trial results, efficacy evaluation methods, and individualized treatment strategies, and summarizing innovative techniques, equipment development, and their integration with other treatment methods.

## Epidemiological basis of CFS

### The global epidemic of CFS

Chronic fatigue syndrome has shown a widespread epidemic trend worldwide, affecting the health of many people. Recent global estimates indicate that more than 65 million people are affected by CFS, and the disorder has major consequences for immune, nervous, and other body systems (1). A systematic review and meta-analysis of the global prevalence of CFS-like symptoms after COVID-19 reported a combined prevalence of 45.2% at 4 weeks, highlighting CFS as a common manifestation of post-COVID-19 illness (2). Another study of 140 patients with COVID-19 found that 43% of the patients met the criteria for ME/CFS; most were female, and obesity (BMI > 30) and poor functional status were significantly associated with ME/CFS (3).

Studies in different regions have confirmed the widespread prevalence of CFS. In Canada, data from the 2010 nationally representative Community Health Survey indicated that the prevalence of self-reported CFS was 1.4% among people over 12 years of age (4). Globally, the estimated prevalence of CFS is between 0.2% and 0.4%, affecting more than 17 million people, with more than 40000 cases estimated in Spain alone (5). These data show that CFS is a global public health problem that cannot be ignored, and that its epidemic trend has brought great challenges to society and the medical system.

### Analysis of the incidence and prevalence of CFS

The incidence and prevalence of CFS vary across studies and populations. In Canada, the 2010 National Community Health Survey showed that about 0.3% of individuals aged 12 years and older were diagnosed with CFS, ME, or fibromyalgia (4). In a study in the Netherlands, the prevalence of CFS was 1.3%, FM was 3.0%, and irritable bowel syndrome (IBS) was 9.7% in 94,516 participants (6). Studies on specific populations have also reported differences. Among female veterans, the prevalence of chronic multisymptomatic illness (CMI) -related diagnoses was 8.2%, which was much higher than the prevalence of 3.9% among all female veterans (7). In a cross-sectional study of Chinese men over 45 years of age, approximately 30% of the participants experienced fatigue, and the proportion of those who met the diagnostic criteria for CFS was not explicitly mentioned; however, the study revealed the prevalence of CFS in men of specific ages (8). These differences may be related to differences in the study methods, diagnostic criteria, and population characteristics, further emphasizing the complexity of accurately assessing the incidence and prevalence of CFS.

### Risk factors and susceptible population of CFS

The occurrence of CFS is associated with multiple risk factors, and specific populations show higher susceptibility. Studies have shown that some genetic polymorphisms may be associated with CFS susceptibility. For example, in a Norwegian study, 427 patients with CFS and healthy controls were analyzed, and some single-nucleotide polymorphisms (SNPs) in the TPPP gene region were found to be associated with CFS, but they did not reach the genome-wide significance level (9).

Infection is considered to be an important trigger in terms of environmental factors. For example, Epstein-Barr virus infection is associated with the development of CFS, and some patients develop chronic fatigue symptoms after infection (10). Childhood trauma is also a known risk factor. In a study of 166 CFS patients, 55 had childhood trauma, in which emotional neglect and emotional abuse were more common, and the presence of personality disorders (PD) in patients was significantly related to childhood emotional trauma (11).

**FIGURE 1:**
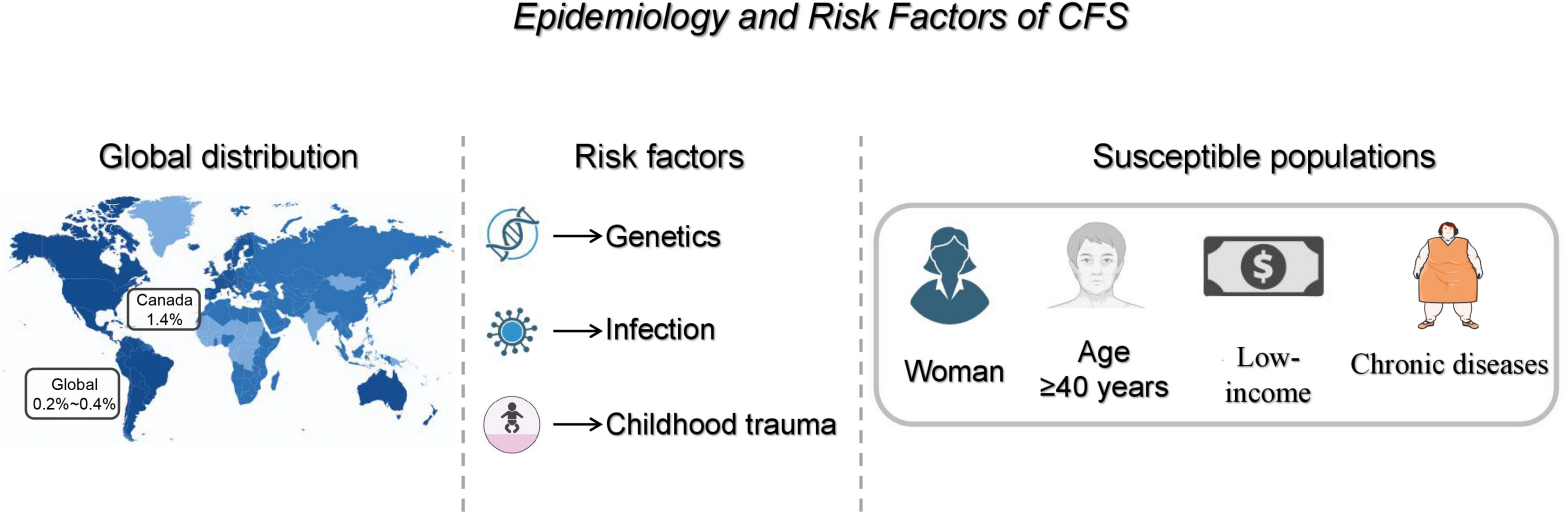
Epidemiology and risk factors of CFS. Schematic overview of the global prevalence of CFS and the main associated risk factors, including genetic susceptibility, prior infections, and childhood trauma. The figure also highlights key high-risk groups (women, individuals ≥40 years, low socioeconomic status, and people with chronic comorbidities). CFS, chronic fatigue syndrome. Created with BioRender.com.

The prevalence of CFS is relatively high in women, adults over 40 years of age, low-income individuals, and individuals with certain chronic disease risk factors (such as obesity, physical inactivity, and smoking) (4, 12). For example, in a study of middle-aged and elderly women in the community, the prevalence of fatigue in women aged 45 years and older was 33.9%, and multivariate logistic regression analysis showed that factors such as older age, single sex, lower education, presence of chronic diseases, underweight, hospitalization in the last year, postmenopause, and more births were associated with an increased risk of fatigue (13). These findings can help to identify high-risk individuals and provide evidence for early prevention and intervention (Figure 1).

## Diagnostic techniques for CFS

### The evolution of clinical diagnostic criteria for CFS

The clinical diagnostic criteria for CFS have evolved over a number of stages. The terminology and diagnostic criteria for the syndrome have changed since its formal appearance in medical literature in 1988. In the early stages, different names such as neurasthenia, epidemic neuromyositis, and benign ME gradually developed into the current proposal of post-effort (14).

The International Classification of Diseases (ICD-10) classifies the syndrome as a neurological disorder; however, there is still a lack of clear histopathological findings to elucidate its nature. Many organizations and institutions have proposed different diagnostic criteria, such as the criteria of the Centers for Disease Control and Prevention (CDC) in 1994, which emphasize that persistent or recurrent fatigue lasts for more than six months, and other diseases that may cause fatigue should be excluded (15).

In 2015, the US Institute of Medicine introduced the SEID criteria, which require three core symptoms (i.e., marked reduction in activity, post-exertional malaise, and unrefreshing sleep) plus at least one additional feature, namely cognitive impairment or orthostatic intolerance (16). However, this recommendation has been controversial, the new name has not been adequately reviewed by patients and professionals, and new diagnostic criteria have not been evaluated in patient and control datasets. Some studies have pointed out that the new diagnostic criteria may lead to a significant increase in prevalence, especially due to the vague definition of excluded diseases, which may include more patients with mild symptoms and less functional impairment (17).

### Research progress on biomarkers of CFS

The identification of biomarkers for CFS has been a focus of research. Regarding immune-related aspects, studies have found that CFS patients have abnormalities in immune cells and immune factors. For example, in a retrospective analysis of 300 CFS patients, 15% had mannose-binding lectin (MBL) deficiency compared with 6% in historical controls, suggesting a possible association between MBL deficiency and CFS. Humoral immunodeficiency is more common in patients with CFS and is associated with respiratory infections (18).

Metabolomics analysis of ME/CFS plasma in metabolism-related biomarker studies found that compounds belonging to the lipid fatty acid metabolic sub-pathway, such as acylcholine, were consistently reduced in two different cohorts of ME/CFS patients, while steroid levels were extensively reduced, and reduced dipeptide levels and increased sphingolipid levels were also observed in patients (1, 19). Changes in these metabolites may be associated with multiple organ system symptoms in patients with ME/CFS, providing new clues for the diagnosis and pathogenesis of the disease. However, the results of current metabolomics studies on CFS are inconsistent, and it is difficult to identify specific and consistent biomarkers due to differences in diagnostic criteria, analytical methods, and samples among different studies (20).

### Imaging diagnostic techniques of CFS

Imaging techniques are important tools for the diagnosis and pathogenesis of CFS. Structural and functional magnetic resonance imaging (MRI) studies have found a variety of abnormal changes in the brains of patients with CFS. MRI studies of 25 CFS patients and 25 normal controls found increased T1-weighted signal intensity in the ventrolateral thalamus, internal capsule, and prefrontal white matter with increasing CFS severity, as well as changes in T2-weighted signal intensity in the white matter of the right middle temporal lobe compared with the course of CFS and with normal controls. Abnormal nerve conduction in this region may affect cognitive function (21).

**FIGURE 2:**
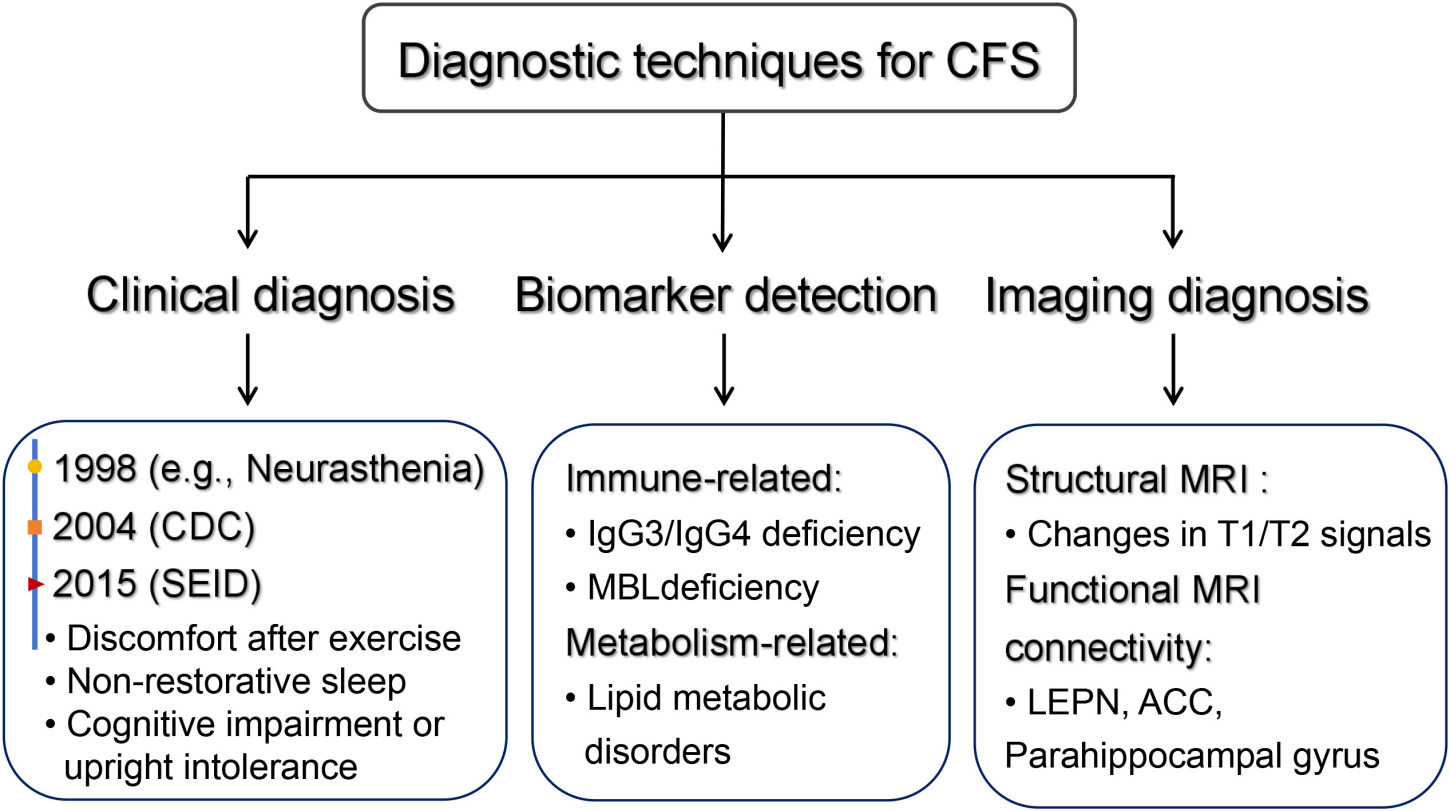
Diagnostic approaches for CFS. Summary of the principal diagnostic modalities for CFS, including clinical criteria (Fukuda 1998, CDC 2004, SEID 2015), candidate immune and metabolic biomarkers, and neuroimaging findings on MRI (white-matter signal changes and altered connectivity in networks such as LEPN and ACC). CFS, chronic fatigue syndrome; CDC, Centers for Disease Control and Prevention; SEID, systemic exertion intolerance disease; MRI, magnetic resonance imaging; LEPN, limbic-emotional processing network; ACC, anterior cingulate cortex.

Functional continuity MRI studies have shown that patients with CFS have altered functional continuity in multiple brain regions at rest. For example, in a study of 36 women, ME/CFS patients were found to have reduced intrinsic continuity of regions within the left frontoparietal network (LEPN), as well as significantly reduced connectivity of the left anterior cingulate with the sensorimotor network (SMN) and the left posterior cingulate with the saliency network (SN) (22). Another functional connectivity analysis based on arterial spin labeling (ASL) found that patients with ME/CFS were in the superior prefrontal gyrus bilaterally. The functional connectivity of the anterior cingulate cortex (ACC), precuneus, and right angular gyrus is equivalent to that of healthy controls, and the connectivity of the left parahippocampal gyrus is closely related to the overall clinical fatigue of patients (23). These imaging changes provide intuitive evidence for further understanding the neural mechanism of CFS and help to further explore the pathophysiological process of the disease (Figure 2).

## Genetics of CFS

### Analysis of genetic susceptibility to CFS

The purpose of genetic susceptibility studies of CFS is to explore the role of genetic factors in the occurrence of the disease. Several studies have analyzed the genes of patients with CFS using different methods and found some genetic variations related to the disease. In a genome-wide association analysis of patients with ME/CFS, DNA from patients and healthy controls was analyzed using chips representing more than 906,600 known SNPs, and 442 candidate SNPs associated with ME/CFS were identified, 12 of which were located in the coding regions of the respective genes. Including one missense substitution in a pattern recognition receptor and one in an uncharacterized coiled-coil domain protein, while also identifying five SNP clusters in the non-coding region of the T-cell receptor locus, these findings provide potential targets for further investigation of the pathophysiological mechanisms of ME/CFS (24).

Another genetic analysis study of 383 ME/CFS participants found that most SNPs were related to the immune system, hormones, metabolism, and extracellular matrix tissue, by screening and functional annotation analysis of gene data from the commercial company 23andMe. A CADD score identified 517 SNPs in these pathways as among the 10% most deleterious substitutions in the human genome, suggesting that these genetic variants may play a role in the morbidity mechanism of ME/CFS (25). In addition, studies have found that the methylation patterns of some specific genes differ in patients with ME/CFS. For example, methylation analysis of peripheral blood mononuclear cell DNA from 10 ME/CFS patients and 10 age/sex-matched healthy controls has found that there are 76 differentially methylated fragments and 394 differentially methylated cytosines in ME/CFS patients. It involves 17 regulatory regions of protein-coding genes related to metabolism and immune activity, and these differences may affect the expression of related genes, thereby participating in the occurrence and development of diseases (26).

### Genomic association study of CFS

Genomic association studies (GWAS) aim to comprehensively identify genetic variants associated with CFS. In a GWAS study of ME/CFS, 427 patients in Norway, 460 patients in Denmark, and 2,105 patients in the UK Biobank were analyzed. Although no ME/CFS risk loci were found to reach genome-wide significance, the TPPP gene region showed the most significant association in the Norwegian discovery cohort. The expression of this gene in the brain tissue suggests that it may be related to the nervous system in the morbidity mechanism of CFS, but this association has not been repeatedly verified in subsequent studies (9).

Another study, through a combined analysis of ME/CFS cohorts in the UK Biobank, found 199 SNPs mapped to 14 genes that were significantly associated with 91% of ME/CFS cases, and these SNPs were grouped into 15 clusters involving multiple genes associated with key cellular mechanisms of CFS. For example, susceptibility to stress and infection, mitochondrial dysfunction, sleep disorders, and autoimmune development have been replicated in the post-viral fatigue syndrome cohort and the fibromyalgia cohort, providing new clues for the genetic mechanism of CFS (27). In addition, the causal relationship between gut microbiota and ME/CFS was explored in a Mendelian randomization study, and the genera *Paraprevotella* and Ruminococcaceae_UCG_014 were found to be positively associated with ME/CFS risk. It has been suggested that gut microbiota may play a role in the pathogenesis of CFS through genetic association (28).

### Study on familial aggregation of CFS

Familial aggregation provides further evidence of the genetic factors of CFS. Some studies have found a certain aggregation phenomenon in the CFS family through the investigation and analysis of family members of patients. In a study of some families of patients with CFS, it was found that the incidence of CFS or related symptoms in relatives of patients was higher than that in the general population, suggesting that genetic factors may play an important role in CFS morbidity (24).

In a study of familial aggregation of multiple chronic diseases, data from the Utah Population Database were analyzed to calculate the relative risks of six diseases, including ME/CFS, in first-, second-, and third-degree relatives. The results showed that all six diseases had significant genetic correlations at all relative levels. Twenty-six of the 30 possible bidirectional disease interrelationships were significant in first-degree relatives, 26 in second-degree relatives, and seven in third-degree relatives, suggesting that ME/CFS may share a common genetic component with other chronic diseases (29). In addition, the study of some specific gene polymorphisms and family clustering of CFS has found that some gene variants may be transmitted in families and increase the risk of disease; however, the specific inheritance pattern and related genes need to be further clarified (30). These studies provide important clues for understanding the morbidity mechanism of CFS from the perspective of family heredity, and are helpful for further targeted genetic research and disease prediction.

## Pathophysiological mechanisms of CFS

### Immune system abnormalities in CFS

Patients with CFS exhibit various abnormalities in the immune system. In terms of immune cells, natural killer (NK) cell function and phenotype were found to be different in patients with CFS. No reproducible differences in NK cell number, cytotoxic granule content, activation status, exocytosis, target cell killing, or cytokine production were observed between 48 patients assessed for ME/CFS in 2003 in Canada and matched healthy controls. However, one patient expressed low levels of perforin due to the p.A91V variant of PRF1, suggesting that NK-cell-related gene variants may be present in some patients (31).

In terms of immunoglobulins and cytokines, a retrospective analysis of 300 patients with CFS showed that 25% of patients had reduced serum levels of at least one antibody class or subclass, with IgG3 and IgG4 subclass deficiency being the most common, whereas increased immunoglobulin levels, especially IgM and IgG2 excess, were observed in another 25% of patients. In addition, MBL deficiency was present in 15% of patients with CFS compared to 6% of historical controls (18). A systematic review and meta-analysis of 42 studies showed that tumor necrosis factor, interleukin-2 (IL-2), IL-4, transforming growth factor-β, and C-reactive protein levels were significantly elevated in patients with CFS, suggesting that activation of the immune system may play an important role in CFS morbidity. However, the inconsistency in the results suggests that inflammation may not be a dominant feature in all patients (32).

### Neuroendocrine disorders in CFS

Neuroendocrine disorders play an important role in CFS pathophysiology. Studies have shown that CFS patients have abnormalities in the hypothalamic-pituitary-adrenal (HPA) axis function. In a study of 21 CFS patients and 20 healthy controls, CFS patients showed increased sensitivity to glucocorticoids (GCs), as assessed by the Trier Social Stress Test (TSST). It was demonstrated that *in vitro* dexamethasone inhibition of lipopolysaccharide (LPS) production of IL-6 and tumor necrosis factor-α (TNF-α) was more pronounced in CFS patients, although there was no significant difference in the cortisol response between the two groups after TSST (33).

In addition, studies in adolescents with CFS found significantly higher plasma levels of norepinephrine, epinephrine, and free thyroxine (FT4) and a significantly lower urinary cortisol/creatinine ratio compared to healthy controls. Network analysis showed a stronger interconnection among immune, nervous, endocrine, and metabolic indicators in adolescents with CFS than in healthy controls, suggesting more pronounced multi-system dysregulation (34). These neuroendocrine abnormalities may affect the body’s stress response and metabolic regulation and then participate in the CFS morbidity mechanism.

### Mechanisms of metabolic abnormalities in CFS

There are several metabolic abnormalities in patients with CFS. In terms of energy metabolism, metabolomic studies in patients with ME/CFS have found abnormalities in multiple metabolic pathways. Analysis of 200 ME/CFS patients and 102 healthy individuals showed that the levels of amino acids that fuel the TCA cycle were reduced in patients, especially in women, whereas the levels of 3-methylhistidine were significantly increased in men, along with increased protein catabolism (35). In addition, analysis of the patient’s peripheral blood mononuclear cells showed increased mRNA expression of pyruvate dehydrogenase (PDH) inhibitory kinases 1, 2, and 4; sirtuin 4; and PPARδ, supporting the hypothesis that PDH function is impaired, which is consistent with the patient’s clinical signs of insufficient ATP production and excessive lactate production by oxidative phosphorylation during exercise.

**FIGURE 3:**
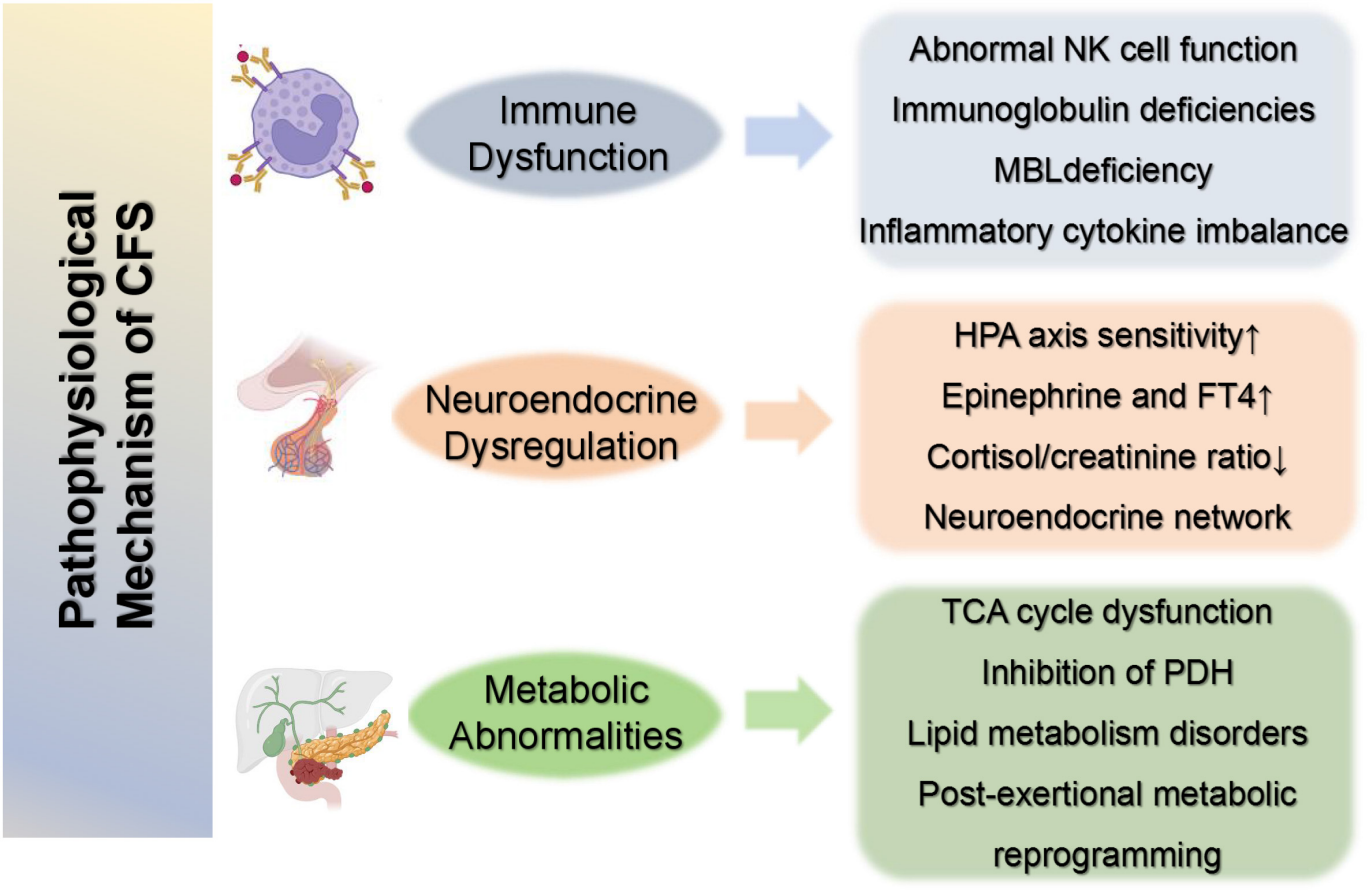
Proposed pathophysiological mechanisms of CFS. Integrated view of the major mechanisms implicated in CFS, encompassing immune dysregulation (NK-cell dysfunction, immunoglobulin/MBL abnormalities, pro-inflammatory cytokines), neuroendocrine disturbance (HPA-axis imbalance, cortisol rhythm changes, FT4 alterations), and metabolic impairment (reduced TCA efficiency, impaired PDH activity, constrained fatty-acid oxidation, and post-exertional energy deficit). CFS, chronic fatigue syndrome; NK, natural killer; MBL, mannose-binding lectin; HPA, hypothalamic–pituitary–adrenal; FT4, free thyroxine; TCA, tricarboxylic acid; PDH, pyruvate dehydrogenase. Created with BioRender.com.

In terms of lipid metabolism, a study of plasma metabolic characteristics in ME/CFS patients found that 74 metabolites were differentially accumulated, of which 35 were still significantly changed after statistical adjustment, involving taurine metabolism, glycerophosphate metabolism, primary bile acid metabolism, purine and pyrimidine metabolism, and other pathways related to fatty acid metabolism. Lipid metabolism disorders may play an important role in the mechanism of CFS morbidity (36). In addition, studies on CFS rat models have found that the TCA cycle, alanine, aspartic acid, glutamate metabolism, and steroid hormone biosynthesis were significantly affected before and after exercise, and sphingolipid metabolism was significantly changed after exercise, indicating that metabolic abnormalities in CFS patients are associated with exercise. This may affect a patient’s exercise endurance and recovery ability (37). These metabolic abnormalities may contribute to fatigue and other symptoms in CFS patients (Figure 3).

## Basic theory of acupuncture and moxibustion treatment of CFS

### Historical application of acupuncture in CFS

Acupuncture is an important therapy in traditional Chinese medicine and has a long history of use in the treatment of CFS. In ancient China, although there is no exact name of “CFS,” similar symptoms have been recorded for a long time. Ancient physicians used acupuncture and moxibustion on specific acupoints to regulate the functions of qi, blood, and viscera and alleviate fatigue symptoms.

In modern research, many clinical observations have verified the effectiveness of acupuncture for the treatment of CFS. For example, 310 patients were included in a study and treated with acupuncture combined with psychotherapy; the clinical survival rate was 88.7% and the effective rate was 97.7%, indicating that acupuncture combined with psychotherapy had a significant effect on CFS (38). Other studies have systematically reviewed the relevant literature on acupuncture and moxibustion treatment of CFS and found that acupuncture and moxibustion improved fatigue symptoms, providing a scientific basis for their clinical application (39).

### Pathogenesis of CFS and acupuncture intervention

The pathological mechanism of CFS is complex and involves the nervous, immune, endocrine, and other systems. Studies have found that patients have abnormalities in the central nervous system, such as structural changes in the frontal cortex and thalamus, abnormalities in factors such as interferon-α (INF-α) and IL-10 in the cerebrospinal fluid, as well as changes in oxidative stress and neurochemicals (40).

Thus, acupuncture may play a therapeutic role by regulating these mechanisms. In a randomized controlled trial (RCT), CFS patients receiving acupuncture aimed at modulating inflammatory pathways showed greater symptom improvement than controls, suggesting that regulation of inflammatory responses may be one mechanism of action (41). Other studies have shown that acupuncture can regulate the autonomic nervous system and improve the heart rate variability of patients, thus alleviating fatigue symptoms. The mechanism may be related to the regulation of the sympathetic and parasympathetic nervous systems by acupuncture at specific acupoints (42).

### Theoretical basis of acupuncture and moxibustion treatment of CFS

Acupuncture treatment for CFS is based on the holistic TCM concept and meridian theory. Traditional Chinese medicine believes that the human body is an organic whole, and the meridians are the channels for the circulation of Qi and blood, connecting the viscera and the body. When Qi and blood are out of balance and meridians are blocked, fatigue and other symptoms occur. Acupuncture can improve CFS symptoms by stimulating acupoints to dredge meridians, harmonize qi and blood, and balance yin and yang.

Modern studies have revealed the underlying mechanisms of acupuncture in the treatment of CFS from the aspects of nerves, immunity, and endocrine function. In terms of nerve regulation, acupuncture may affect the HPA axis and the level of monoamine neurotransmitters to regulate the function of the nervous system (40). In terms of immune regulation, acupuncture can improve the immune status of the body through immune cells and cytokines (43). In addition, acupuncture may play a therapeutic role by regulating energy metabolism and improving intestinal flora (44).

### Application of acupuncture and moxibustion in CFS

Acupuncture is increasingly being used in the treatment of CFS. Several clinical studies have confirmed its effectiveness, such as a multi-center RCT, which divided 150 CFS patients into body acupuncture, Sa-am acupuncture, and control groups. The results showed that the fatigue severity scale (FSS) score of the body acupuncture group was significantly lower than that of the control group after 5 weeks of treatment, indicating that acupuncture may be effective in improving fatigue symptoms in CFS patients (41).

A systematic review and meta-analysis also showed that acupuncture and moxibustion had certain advantages in the treatment of CFS compared to other treatments. For example, a network meta-analysis included 51 RCTs, and the results showed that the total effective rate of acupuncture and moxibustion was higher than that of other treatment methods, and it also showed better performance in improving the fatigue scale (FS-14) score (45). However, the quality of the existing studies is uneven, and some studies have small sample sizes and methodological defects, which affect the reliability of the conclusions.

## Clinical practice of acupuncture and moxibustion in treating CFS

### Clinical trial of acupuncture and moxibustion in CFS

The efficacy of acupuncture in CFS treatment has been verified in several clinical trials. For example, in a multicenter, unblinded RCT, 150 patients with CFS and idiopathic chronic fatigue were divided into a body acupuncture group, Sa-am acupuncture group, and control group. The results showed that after 5 weeks of treatment, the FSS score in the body acupuncture group was significantly lower than that in the control group, and there was a significant improvement in the Stress Reaction Inventory (SRI) and Beck Depression Inventory (BDI) scores. Acupuncture has been suggested to effectively improve fatigue and related symptoms in patients (41).

Another RCT compared the efficacy of moxibustion and acupuncture in the treatment of CFS, and the results showed that the total effective rate of the moxibustion group was 88.9%, which was higher than that of the acupuncture group (72.2%), and the moxibustion group had more advantages in the improvement of fatigue assessment index (FAI) score (46). These studies provide strong clinical evidence for acupuncture treatment for CFS.

### Evaluation of the therapeutic effect of acupuncture and moxibustion on CFS

The fatigue scale, quality of life scale, and related laboratory indicators are often used to evaluate the efficacy of acupuncture in CFS treatment. Fatigue scales, such as the FSS and FS-14, can directly reflect changes in the fatigue degree of patients. Quality of life scales, such as the MOS 36-item Short Form Health Survey (SF-36), can comprehensively assess the improvement of quality of life in patients.

In a systematic review and meta-analysis, 16 studies with a total of 1346 patients were included, and the results showed that acupuncture was superior to sham acupuncture and traditional Chinese medicine in terms of overall effective rate, and could significantly reduce the severity of fatigue (47). Another network meta-analysis showed that moxibustion and traditional Chinese medicine performed well in the total effective rate, and moxibustion plus acupuncture improved the improvement of the FS-14 total score (45).

### Individualized treatment strategy for CFS with acupuncture quality

Acupuncture and moxibustion quality CFS emphasize individualized treatment, which is based on syndrome differentiation and treatment according to the constitution, symptoms, and signs of patients. For example, for CFS patients with spleen deficiency, Pishu, Zusanli and other acupoints can be selected to invigorate the spleen and replenish qi; For patients with kidney deficiency, Shenshu, Guanyuan and other acupoints can be added to tonify the kidney and replenish essence.

**FIGURE 4:**
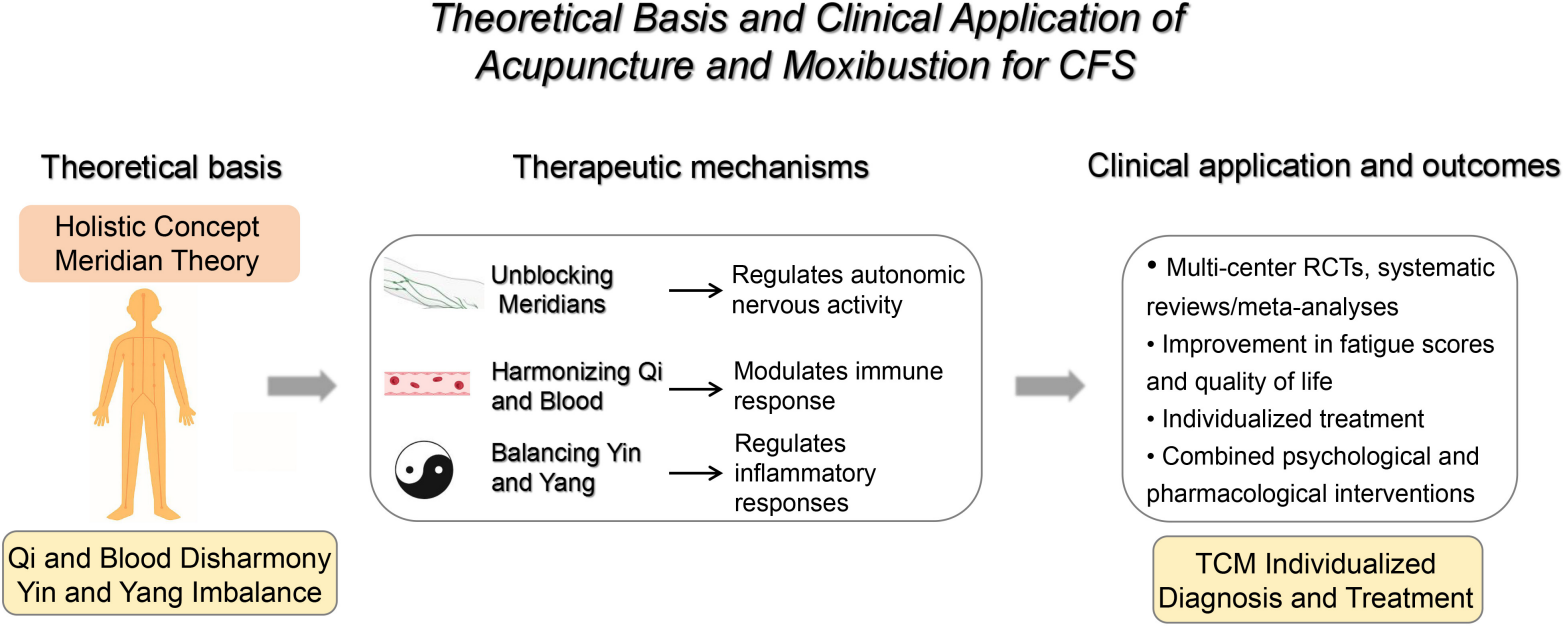
Theoretical framework and clinical application of acupuncture and moxibustion for CFS. Illustration of how TCM concepts (holism, meridians, regulation of qi and blood, yin–yang balance) link to putative neuro-immune–endocrine mechanisms of acupuncture and moxibustion, and to clinical evidence from randomized trials and systematic reviews showing improvements in fatigue severity and quality of life. CFS, chronic fatigue syndrome; TCM, traditional Chinese medicine; RCTs, randomized controlled trials. Created with BioRender.com.

In a study, 40 rats with chronic fatigue were divided into two groups: acupuncture at the Pishu point combined with ginsenoside Rg3, acupuncture, ginsenoside Rg3, and blank control treatment. The results showed that acupuncture at the Pishu point combined with ginsenoside Rg3 had significantly better effects than other groups in improving rat weight and correcting immune imbalance. It has been suggested that individualized acupuncture and moxibustion combination therapy can improve CFS symptoms (48). In addition, according to the specific symptoms of patients, such as insomnia and anxiety, specific acupoints can be added to improve the pertinence and effectiveness of treatment (Figure 4).

## Technical progress of acupuncture and moxibustion in treatment of CFS

### Innovative application of acupuncture and moxibustion techniques in CFS

In recent years, there have been new and innovative applications of acupuncture techniques in the treatment of CFS. For example, electroacupuncture enhances the stimulation intensity and regulation of acupuncture by applying current to acupoints. Studies have found that electroacupuncture stimulation of specific acupoints can regulate the excitability of the cerebral cortex and improve fatigue symptoms in patients with CFS. In a study of 72 CFS patients, electroacupuncture produced greater reductions in fatigue and more significant improvements in cerebral cortical function than routine needling (49).

In addition, acupoint catgut embedding therapy embeds absorbable thread into acupoints and plays a therapeutic role by continuously stimulating acupoints. This therapy has shown potential in the treatment of CFS, which can regulate the immune function of the body and improve fatigue symptoms. For example, in one study, 60 CFS patients with spleen-kidney Yang deficiency were treated with acupoint catgut embedding combined with ginger-partitioned moxibustion. The results showed that the clinical signs and symptoms of the patients were significantly improved, and the activity of T lymphocyte subsets and natural killer cells was regulated (50).

### Development of equipment and tools for acupuncture and moxibustion treatment of CFS

With the development of science and technology, the equipment and tools for the acupuncture treatment of CFS are constantly improving. New acupuncture and moxibustion tools are more humanized in material and design, and improve the safety and comfort of acupuncture. For example, some needles use a thinner body, which reduces pain in patients.

At the same time, the function of electroacupuncture therapeutic instruments, which can accurately adjust the frequency, intensity, and waveform of current to meet the treatment needs of different patients, is becoming increasingly perfect. In addition, there are some auxiliary equipment, such as moxibustion boxes and needle warmers, which make moxibustion operation more convenient and safe and improve patient compliance. The development of these devices and tools provides better technical support for acupuncture treatment of CFS, helps to improve the therapeutic effect, and promotes its application.

### Combined application of acupuncture and moxibustion therapy and other treatment methods

Acupuncture and moxibustion combined with other treatments can play a synergistic role in improving the therapeutic effects of CFS. If acupuncture and moxibustion are combined with traditional Chinese medicine, traditional Chinese medicine can regulate human body function as a whole, and acupuncture and moxibustion can enhance the efficacy of drugs by stimulating acupoints. In one study, 60 patients with CFS were treated with Chaihu Longgu Muli Decoction combined with acupuncture at back-shu points and traditional Chinese medicine alone. The results showed that the improvement in fatigue and anxiety scales in the combined treatment group was better than that in the traditional Chinese medicine alone group, indicating that the combination of acupuncture and traditional Chinese medicine could better alleviate the symptoms of fatigue and anxiety in patients (51).

In addition, acupuncture and moxibustion can be combined with psychotherapy and exercise. Psychotherapy can help patients relieve their mental stress and improve their psychological state. Exercise therapy can enhance physical fitness and improve body functions. Acupuncture combined with these therapies can provide a more comprehensive treatment plan for CFS patients and hopefully achieve better clinical results by intervening in many aspects of the body and mind.

## Controversies and challenges in acupuncture treatment of CFS

### Methodological quality and limitations of current evidence

Although multiple randomized controlled trials and meta-analyses suggest that acupuncture and moxibustion may alleviate fatigue and improve quality of life in patients with CFS, the overall strength of evidence remains limited. Most existing RCTs are single-center studies with relatively small sample sizes and short treatment or follow-up periods, which reduces statistical power and makes it difficult to assess the durability of the reported benefits (41, 45–47). In addition, blinding is often incomplete; for instance, several trials are open-label, and even when sham acupuncture is used, the control procedures and their credibility are not always clearly described, leading to potential performance and detection bias. In addition, the diagnostic criteria and inclusion standards for CFS are heterogeneous across studies (such as Fukuda-based definitions, CDC criteria, or locally modified schemes), and syndrome differentiation in traditional Chinese medicine is not standardized, which further increases clinical heterogeneity. Outcome measures also vary widely, including different fatigue scales (such as FSS, FS-14, and FAI), composite “total effective rate” indices, and diverse quality-of-life instruments, limiting direct comparability between trials and complicating quantitative synthesis. Consistent with these issues, the systematic reviews and network meta-analyses cited in this study have generally rated the certainty of evidence as low to very low, largely due to unclear randomization and allocation concealment, insufficient reporting of protocol registration and primary outcomes, and a potential risk of publication bias (45–47).

Moreover, these methodological concerns help explain why the published literature does not present a uniformly positive picture of acupuncture and moxibustion for CFS. While several trials and pooled analyses report clear advantages over control conditions for global “effective rate” and improvements in fatigue scores, others show only modest benefits, gains restricted to specific subscales, or no statistically significant difference compared with sham acupuncture or non-acupuncture therapies (41, 45–47). In some meta-analyses, sensitivity analyses and subgroup comparisons reveal that the direction and magnitude of effect estimates can shift when trials at higher risk of bias are excluded, when different diagnostic criteria are applied, or when alternative outcome definitions are used, suggesting that part of the apparent benefit may be driven by study design rather than a stable underlying effect. From a clinical perspective, this pattern indicates that acupuncture and moxibustion are promising but not definitively established interventions for CFS, as although they may offer meaningful symptom relief for some patients, particularly when integrated into individualized, multimodal care, current evidence does not justify viewing them as uniformly effective or universally applicable. From a research standpoint, these inconsistencies underline the need for rigorously designed, adequately powered multicenter trials with standardized diagnostic criteria, carefully chosen and transparently reported core outcome sets, and robust control conditions, so that future findings can more reliably guide clinical decision-making and guideline development.

### Safety of acupuncture in the treatment of CFS

The safety of acupuncture treatment for CFS is generally good; however, there are still some potential risks. Local pain, bleeding, infection, and other adverse reactions may occur during acupuncture, especially when acupoints are improperly selected or the operation is not standardized.

In a study of acupuncture for dry eye, five patients experienced acupuncture-related adverse events with relatively mild symptoms (52). Additionally, moxibustion may cause skin burns, allergies, and other problems. Although serious adverse reactions are rare, they require further attention. Clinicians should strictly grasp the indications and contraindications of acupuncture and moxibustion and standardize the surgical process to ensure patient safety.

### Ethical considerations of acupuncture therapy in CFS

Ethical considerations are critical in the treatment of CFS with acupuncture. First, regarding the patient’s right to know, doctors should fully explain the principle, method, possible efficacy, and risk of acupuncture treatment to patients, to ensure that patients receive treatment with informed consent.

Second, in the research process, ethical principles should be followed to protect the privacy and rights of patients. For example, clinical trials should be designed and implemented in strict accordance with relevant ethical norms to ensure the scientific and ethical nature of the study. In addition, acupuncture and moxibustion treatment should avoid overtreatment, choose reasonable acupoints and treatment programs to reduce the pain and economic burden of patients, and reflect the humanistic care of medical treatment.

## Future prospects of acupuncture treatment for CFS

### The research direction of acupuncture and moxibustion in CFS

Future research on acupuncture and moxibustion for CFS should place equal emphasis on high-quality RCTs and well-designed mechanistic studies. From a clinical perspective, upcoming RCTs should follow the principles of randomization, allocation concealment, and, where feasible, blinding of patients and assessors. Multicenter designs with adequately powered sample sizes are needed to reduce single-center bias and improve generalizability. Diagnostic criteria and inclusion standards should be clearly defined and unified (for instance, using established CFS/ME criteria that are consistently applied across sites), and detailed reporting of TCM syndrome differentiation is encouraged to allow meaningful subgroup analyses. Treatment protocols should specify a core set of acupoints and treatment frequency, with transparent rules for individualization, and use well-described control conditions (such as sham acupuncture, usual care, or other active comparators). In terms of outcomes, trials should predefine a limited number of primary and secondary endpoints, including validated fatigue scales (e.g., FSS or FS-14), quality-of-life measures (such as SF-36 where appropriate), and psychological indices, and should incorporate follow-up assessments extending beyond the end of treatment to evaluate the durability of clinical benefits and monitor safety.

**FIGURE 5:**
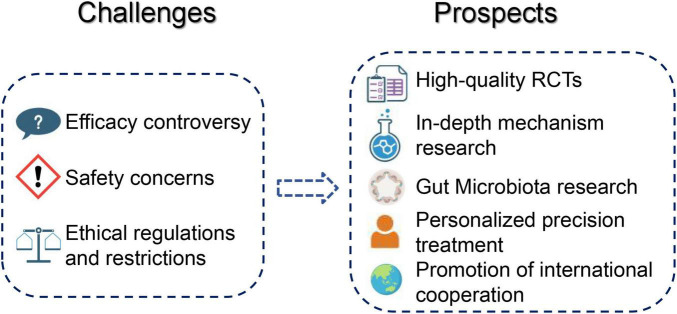
Challenges and future directions for acupuncture and moxibustion in CFS. Overview of current controversies and unmet needs, including methodological limitations of existing trials, safety and ethical considerations, and priority areas for future work such as high-quality RCTs, mechanistic studies (including gut microbiota), personalized treatment strategies, and international collaborative research. CFS, chronic fatigue syndrome; RCTs, randomized controlled trials. Created with BioRender.com.

Mechanistic studies should be more closely integrated with clinical research to clarify how acupuncture and moxibustion act on the nervous, immune, endocrine, and metabolic systems implicated in CFS. Based on current evidence, priority directions include combining neuroimaging techniques (i.e., structural and functional MRI) with clinical outcomes to explore changes in brain networks; profiling immune function by assessing NK-cell activity, immunoglobulin subclasses, MBL levels, and key cytokines; and applying proteomics and metabolomics to characterize shifts in energy and lipid metabolism before and after treatment. Where possible, these mechanistic assessments can be embedded within RCTs as predefined sub-studies, so that changes in imaging or laboratory markers can be directly correlated with symptom improvement. In addition, building on emerging work on gut microbiota in CFS, future studies may examine whether acupuncture and moxibustion modify intestinal flora and related metabolites, and how such changes relate to fatigue and other clinical features. Through this combination of rigorous trial design and targeted mechanistic exploration, future research would be able to provide more solid evidence for the efficacy and mode of action of acupuncture and moxibustion in CFS and better inform clinical and guideline decisions (Figure 5).

### A potential breakthrough in acupuncture treatment of CFS

In terms of potential breakthroughs, new targets and approaches to acupuncture treatment are expected to be found with an in-depth understanding of the pathological mechanism of CFS. For example, the study of intestinal flora is closely related to the occurrence and development of CFS, and acupuncture may play a therapeutic role by regulating intestinal flora and its metabolites, which provides a new research direction for acupuncture treatment of CFS (44).

Additionally, personalized precision treatment is a potential breakthrough. Through a comprehensive analysis of the patients’ genes, physique, lifestyle, and other factors, a more accurate acupuncture treatment plan was formulated to improve the therapeutic effect. Simultaneously, combined with modern science and technology, research and development of more efficient, convenient, and safe acupuncture equipment and technology will also bring new opportunities for acupuncture treatment of CFS.

### Opportunities for international collaboration on acupuncture in CFS

International collaboration provides a pathway to strengthen the evidence base for acupuncture and moxibustion in CFS and to enhance their global relevance. One practical approach would be to establish joint international registries that enroll CFS patients undergoing acupuncture and moxibustion in different regions, using shared case report forms, harmonized diagnostic criteria (e.g., Fukuda, CDC, or SEID), and common outcome measures such as FSS, FS-14, and SF-36. Such registries could capture real-world data on patterns of point selection, treatment frequency, and safety events, and would allow comparative analyses across health-care systems. In addition, collaborative groups could work toward harmonized acupuncture protocols for CFS. For instance, specifying a set of basic acupoints with clearly defined options for syndrome-based individualization could allow clinical investigations conducted in China, Europe, and other regions to be more directly compared or pooled.

Moreover, multinational RCT initiatives represent another important direction. Coordinated phase II/III trials using shared protocols, centralized randomization, and pre-specified core outcomes could provide more robust estimates of efficacy and safety than single-center studies alone. These clinical networks could be linked to joint mechanistic platforms, incorporating neuroimaging, immune profiling, metabolomics, and gut microbiota analyses to explore how acupuncture interventions modulate the nervous, immune, endocrine, and metabolic systems described earlier in this review. Furthermore, international academic exchanges and training programs, such as short-term fellowships, joint workshops on trial methodology, and consensus meetings on reporting standards, would help align research practice, enhance mutual understanding of TCM-based approaches, and support the development of globally applicable guidelines for acupuncture and moxibustion in CFS.

## References

[1] Germain A Barupal DK Levine SM Hanson MR. Comprehensive circulatory metabolomics in ME/CFS reveals disrupted metabolism of Acyl lipids and steroids. *Metabolites*. (2020) 10:34. 10.3390/metabo10010034 31947545 PMC7023305

[2] Salari N Khodayari Y Hosseinian-Far A Zarei H Rasoulpoor S Akbari H Global prevalence of chronic fatigue syndrome among long COVID-19 patients: a systematic review and meta-analysis. *Biopsychosoc Med*. (2022) 16:21. 10.1186/s13030-022-00250-5 36274177 PMC9589726

[3] Bonilla H Quach TC Tiwari A Bonilla AE Miglis M Yang PC Myalgic Encephalomyelitis/Chronic Fatigue Syndrome is common in post-acute sequelae of SARS-CoV-2 infection (PASC): results from a post-COVID-19 multidisciplinary clinic. *Front Neurol*. (2023) 14:1090747. 10.3389/fneur.2023.1090747 36908615 PMC9998690

[4] Rusu C Gee ME Lagacé C Parlor M. Chronic fatigue syndrome and fibromyalgia in Canada: prevalence and associations with six health status indicators. *Health Promot Chronic Dis Prev Can*. (2015) 35:3–11. 10.24095/hpcdp.35.1.02 25811400 PMC4939456

[5] De Vera Martín A Salazar AD Pérez IMM Pérez SEM. Effectiveness of exercise-based rehabilitation in chronic fatigue syndrome: a systematic review and meta-analysis. *Int J Exerc Sci*. (2025) 18:495–530. 10.70252/DAYA4589 40485841 PMC12143281

[6] Janssens KA Zijlema WL Joustra ML Rosmalen JG. Mood and anxiety disorders in chronic fatigue syndrome, fibromyalgia, and irritable bowel syndrome: results from the lifelines cohort study. *Psychosom Med*. (2015) 77:449–57. 10.1097/PSY.0000000000000161 25768845

[7] Mohanty AF Muthukutty A Carter ME Palmer MN Judd J Helmer D Chronic multisymptom illness among female Veterans deployed to Iraq and Afghanistan. *Med Care*. (2015) 53(4 Suppl 1):S143–8. 10.1097/MLR.0000000000000314 25767968

[8] Lin WQ Jing MJ Tang J Wang JJ Zhang HS Yuan LX Factors associated with fatigue among men aged 45 and older: a cross-sectional study. *Int J Environ Res Public Health*. (2015) 12:10897–909. 10.3390/ijerph120910897 26404346 PMC4586650

[9] Hajdarevic R Lande A Mehlsen J Rydland A Sosa DD Strand EB Genetic association study in myalgic encephalomyelitis/chronic fatigue syndrome (ME/CFS) identifies several potential risk loci. *Brain Behav Immun*. (2022) 102:362–9. 10.1016/j.bbi.2022.03.010 35318112

[10] Ruiz-Pablos M Paiva B Montero-Mateo R Garcia N Zabaleta A. Epstein-barr virus and the origin of myalgic encephalomyelitis or chronic fatigue syndrome. *Front Immunol*. (2021) 12:656797. 10.3389/fimmu.2021.656797 34867935 PMC8634673

[11] Sáez-Francàs N Calvo N Alegre J Castro-Marrero J Ramírez N Hernández-Vara J Childhood trauma in Chronic Fatigue Syndrome: focus on personality disorders and psychopathology. *Compr Psychiatry*. (2015) 62:13–9. 10.1016/j.comppsych.2015.06.010 26343462

[12] Arron HE Marsh BD Kell DB Khan MA Jaeger BR Pretorius E. Myalgic encephalomyelitis/chronic fatigue syndrome: the biology of a neglected disease. *Front Immunol*. (2024) 15:1386607. 10.3389/fimmu.2024.1386607 38887284 PMC11180809

[13] Jing MJ Wang JJ Lin WQ Lei YX Wang PX. A community-based cross-sectional study of fatigue in middle-aged and elderly women. *J Psychosom Res*. (2015) 79:288–94. 10.1016/j.jpsychores.2015.05.009 26028605

[14] Murga Í Lafuente JV. [From neurasthenia to post-exertion disease: evolution of the diagnostic criteria of chronic fatigue syndrome/myalgic encephalomyelitis]. *Aten Primaria.* (2019) 51:579–85. 10.1016/j.aprim.2019.04.004 31182238 PMC6945124

[15] Larun L Brurberg KG Odgaard-Jensen J Price JR. Exercise therapy for chronic fatigue syndrome. *Cochrane Database Syst Rev*. (2019) 10:CD003200. 10.1002/14651858.CD003200.pub8 31577366 PMC6953363

[16] Larrimore C Ramnot A Jaghab A Sarduy S Guerrero G Troccoli P Understanding myalgic encephalomyelitis/chronic fatigue syndrome and the emerging osteopathic approach: a narrative review. *J Am Osteopath Assoc*. (2019) 119:446–55. 10.7556/jaoa.2019.081 31233110

[17] Jason LA Sunnquist M Brown A McManimen S Furst J. Reflections on the Institute of Medicine’s systemic exertion intolerance disease. *Pol Arch Med Wewn*. (2015) 125:576–81. 10.20452/pamw.2973 26176405 PMC4826027

[18] Guenther S Loebel M Mooslechner AA Knops M Hanitsch LG Grabowski P Frequent IgG subclass and mannose binding lectin deficiency in patients with chronic fatigue syndrome. *Hum Immunol*. (2015) 76:729–35. 10.1016/j.humimm.2015.09.028 26429318

[19] López-Amador N. *Orexinergic and Hypothalamic Dysfunction in Chronic Fatigue Syndrome: A Mechanistic Framework for Biomarker Discovery and Targeted Therapies.* (2025). Available online at: https://www.qeios.com/read/ZOZBH7/pdf [Accessed February 17, 2025].

[20] Huth TK Eaton-Fitch N Staines D Marshall-Gradisnik S. A systematic review of metabolomic dysregulation in Chronic Fatigue Syndrome/Myalgic Encephalomyelitis/Systemic Exertion Intolerance Disease (CFS/ME/SEID). *J Transl Med*. (2020) 18:198. 10.1186/s12967-020-02356-2 32404171 PMC7222338

[21] Barnden LR Crouch B Kwiatek R Burnet R Del Fante P. Evidence in chronic fatigue syndrome for severity-dependent upregulation of prefrontal myelination that is independent of anxiety and depression. *NMR Biomed*. (2015) 28:404–13. 10.1002/nbm.3261 25702943 PMC4369127

[22] Gay CW Robinson ME Lai S O’Shea A Craggs JG Price DD Abnormal resting-state functional connectivity in patients with chronic fatigue syndrome: results of seed and data-driven analyses. *Brain Connect*. (2016) 6:48–56. 10.1089/brain.2015.0366 26449441 PMC4744887

[23] Boissoneault J Letzen J Lai S O’Shea A Craggs J Robinson ME Abnormal resting state functional connectivity in patients with chronic fatigue syndrome: an arterial spin-labeling fMRI study. *Magn Reson Imaging*. (2016) 34:603–8. 10.1016/j.mri.2015.12.008 26708036 PMC4801728

[24] Schlauch KA Khaiboullina SF De Meirleir KL Rawat S Petereit J Rizvanov AA Genome-wide association analysis identifies genetic variations in subjects with myalgic encephalomyelitis/chronic fatigue syndrome. *Transl Psychiatry*. (2016) 6:e730. 10.1038/tp.2015.208 26859813 PMC4872418

[25] Perez M Jaundoo R Hilton K Del Alamo A Gemayel K Klimas NG Genetic predisposition for immune system, hormone, and metabolic dysfunction in myalgic encephalomyelitis/chronic fatigue syndrome: a pilot study. *Front Pediatr*. (2019) 7:206. 10.3389/fped.2019.00206 31179255 PMC6542994

[26] Helliwell AM Sweetman EC Stockwell PA Edgar CD Chatterjee A Tate WP. Changes in DNA methylation profiles of myalgic encephalomyelitis/chronic fatigue syndrome patients reflect systemic dysfunctions. *Clin Epigenetics*. (2020) 12:167. 10.1186/s13148-020-00960-z 33148325 PMC7641803

[27] Das S Taylor K Kozubek J Sardell J Gardner S. Genetic risk factors for ME/CFS identified using combinatorial analysis. *J Transl Med*. (2022) 20:598. 10.1186/s12967-022-03815-8 36517845 PMC9749644

[28] He G Cao Y Ma H Guo S Xu W Wang D Causal effects between gut microbiome and myalgic encephalomyelitis/chronic fatigue syndrome: a two-sample Mendelian randomization study. *Front Microbiol*. (2023) 14:1190894. 10.3389/fmicb.2023.1190894 37485509 PMC10359717

[29] Allen-Brady K Fyer AJ Weissman M. The multi-generational familial aggregation of interstitial cystitis, other chronic nociplastic pain disorders, depression, and panic disorder. *Psychol Med*. (2023) 53:7847–56. 10.1017/S0033291723001885 37458197

[30] Marathe CS Torpy DJ. A role for corticosteroid-binding globulin variants in stress-related disorders. *Expert Rev Endocrinol Metab*. (2012) 7:301–8. 10.1586/eem.12.20 30780848

[31] Theorell J Bileviciute-Ljungar I Tesi B Schlums H Johnsgaard MS Asadi-Azarbaijani B Unperturbed cytotoxic lymphocyte phenotype and function in myalgic encephalomyelitis/chronic fatigue syndrome patients. *Front Immunol*. (2017) 8:723. 10.3389/fimmu.2017.00723 28694809 PMC5483846

[32] Strawbridge R Sartor ML Scott F Cleare AJ. Inflammatory proteins are altered in chronic fatigue syndrome-A systematic review and meta-analysis. *Neurosci Biobehav Rev*. (2019) 107:69–83. 10.1016/j.neubiorev.2019.08.011 31465778

[33] Gaab J Rohleder N Heitz V Schad T Engert V Schürmeyer TH Enhanced glucocorticoid sensitivity in patients with chronic fatigue syndrome. *Acta Neuropsychiatr*. (2003) 15:184–91. 10.1034/j.1601-5215.2003.00033.x 26983566

[34] Wyller VB Vitelli V Sulheim D Fagermoen E Winger A Godang K Altered neuroendocrine control and association to clinical symptoms in adolescent chronic fatigue syndrome: a cross-sectional study. *J Transl Med*. (2016) 14:121. 10.1186/s12967-016-0873-1 27149955 PMC4858924

[35] Fluge Ø Mella O Bruland O Risa K Dyrstad SE Alme K Metabolic profiling indicates impaired pyruvate dehydrogenase function in myalgic encephalopathy/chronic fatigue syndrome. *JCI Insight.* (2016) 1:e89376. 10.1172/jci.insight.89376 28018972 PMC5161229

[36] Germain A Ruppert D Levine SM Hanson MR. Metabolic profiling of a myalgic encephalomyelitis/chronic fatigue syndrome discovery cohort reveals disturbances in fatty acid and lipid metabolism. *Mol Biosyst*. (2017) 13:371–9. 10.1039/c6mb00600k 28059425 PMC5365380

[37] Shao C Ren Y Wang Z Kang C Jiang H Chi A. Detection of urine metabolites in a rat model of chronic fatigue syndrome before and after exercise. *Biomed Res Int*. (2017) 2017:8182020. 10.1155/2017/8182020 28421200 PMC5380834

[38] Guo J. Chronic fatigue syndrome treated by acupuncture and moxibustion in combination with psychological approaches in 310 cases. *J Tradit Chin Med.* (2007) 27:92–5. 17710799

[39] Feng C Qu Y Lu J Guo S Li B Shao Y Review of the research progress and future prospects of acupuncture in the treatment of chronic fatigue syndrome. *Holist Nurs Pract*. (2025) 39:335–45. 10.1097/HNP.0000000000000727 40245265

[40] Li BB Feng CW Qu YY Sun ZR Chen T Wang YL Research progress on central mechanism of acupuncture treatment for chronic fatigue syndrome. *World J Acupunct Moxibustion.* (2023) 10.1016/j.wjam.2023.03.002. [Online ahead of print]. 37363406 PMC10061266

[41] Kim JE Seo BK Choi JB Kim HJ Kim TH Lee MH Acupuncture for chronic fatigue syndrome and idiopathic chronic fatigue: a multicenter, nonblinded, randomized controlled trial. *Trials*. (2015) 16:314. 10.1186/s13063-015-0857-0 26211002 PMC4515016

[42] Li T Litscher G Zhou Y Song Y Shu Q Chen L Effects of acupuncture and moxibustion on heart rate variability in chronic fatigue syndrome patients: regulating the autonomic nervous system in a clinical randomized controlled trial. *Complement Ther Med*. (2025) 92:103184. 10.1016/j.ctim.2025.103184 40315935

[43] Wang XY Liu CZ Lei B. [Effect of acupuncture on the expression of transcription factor T-bet/GATA-3 in plasma of rats with chronic fatigue syndrome]. *Zhen Ci Yan Jiu.* (2017) 42:246–8. 29071982

[44] Li CR Sun ZR Wang YL Yang Y Sun WB Qu YY [Mechanism of acupuncture and moxibustion in treatment of chronic fatigue syndrome from perspective of intestinal flora]. *Zhongguo Zhen Jiu*. (2022) 42:956–60. 10.13703/j.0255-2930.20210829-k0003 35938342

[45] Fang Y Yue BW Ma HB Yuan YP. Acupuncture and moxibustion for chronic fatigue syndrome: a systematic review and network meta-analysis. *Medicine*. (2022) 101:e29310. 10.1097/MD.0000000000029310 35945779 PMC9351926

[46] Tian L Wang J Luo C Sun R Zhang X Yuan B [Moxibustion at Gaohuang (BL 43) for chronic fatigue syndrome: a randomized controlled trial]. *Zhongguo Zhen Jiu.* (2015) 35:1127–30. 26939325

[47] Zhang Q Gong J Dong H Xu S Wang W Huang G. Acupuncture for chronic fatigue syndrome: a systematic review and meta-analysis. *Acupunct Med*. (2019) 37:211–22. 10.1136/acupmed-2017-011582 31204859

[48] Zhang W Zhang Y Ma X Chen Y. Effects of acupuncturing Pishu combined with Ginsenoside Rg3 on the immune function of rats with chronic fatigue. *Int J Clin Exp Med.* (2015) 8:19022–9. 26770528 PMC4694428

[49] Li ZX Zhang Y Yan LD Lai MQ Xu HY Wu T [Effect of electroacupuncture at back- shu points of five zang on fatigue status and cortical excitability in chronic fatigue syndrome]. *Zhongguo Zhen Jiu*. (2022) 42:1205–10. 10.13703/j.0255-2930.20220124-k0006 36397215

[50] Xia D Chen P Du P Ding L Liu A. [Efficacy of acupoint catgut embedding combined with ginger-partitioned moxibustion on chronic fatigue syndrome of spleen-kidney yang deficiency syndrome and its effects on T lymphocyte subsets and activity of NK cell]. *Zhongguo Zhen Jiu*. (2017) 37:814–8. 10.13703/j.0255-2930.2017.08.004 29231339

[51] Qi Y Song S Dou Z Chen J He G Zhang L [Chaihu Longgu Muli decoction combined with acupuncture at back- shu points for chronic fatigue syndrome]. *Zhongguo Zhen Jiu*. (2017) 37:1187–90. 10.13703/j.0255-2930.2017.11.013 29354956

[52] Zhang X Zhang B Peng S Zhang G Ma J Zhu W. Effectiveness of acupuncture at acupoint BL1 (Jingming) in comparison with artificial tears for moderate to severe dry eye disease: a randomized controlled trial. *Trials*. (2022) 23:605. 10.1186/s13063-022-06486-4 35897025 PMC9327344

