# Supplementary material for: From tradition to healing: the promise of acupuncture in managing chronic fatigue syndrome
Source: Front Med (Lausanne). 2026 Jan 20;12:1724290. doi: 10.3389/fmed.2025.1724290 (PMC12865710; doi:10.3389/fmed.2025.1724290)
Supplement: Supplementary file 1 [file Table_1.docx]

**Supplementary Material for**

**From Tradition to Healing: The Promise of Acupuncture in Managing Chronic Fatigue Syndrome**

Delong Wang^1^ Tiansong Yang^2^ Yang Cui^3^ Yuanyuan Qu^2^ Chuwen Feng^2^ Zhongren Sun^4^ Miao Zhang^5*^

1. Key Laboratory of Neurobiology of Clinical Acupuncture, The Second Affiliated Hospital of Heilongjiang University of Chinese Medicine, Harbin, Heilongjiang Province, 150040, China

2. Rehabilitation Department II, The First Affiliated Hospital of Heilongjiang University of Chinese Medicine, Harbin, Heilongjiang Province,150040, China

3. Acupuncture Department Ⅶ, The Second Affiliated Hospital of Heilongjiang University of Chinese Medicine, Harbin, Heilongjiang, 150001, China

4. Heilongjiang University of Chinese Medicine, Harbin, Heilongjiang Province,150040, China

5. The Second Affiliated Hospital of Heilongjiang University of Chinese Medicine, Harbin, Heilongjiang Province, 150040, China

*Correspondence: No. 411, Gogol Street, Nangang District, Harbin, Heilongjiang Province, 150001, China

**Methods**

**Literature search strategy**

This narrative review was designed based on a structured search of both international and Chinese databases, including PubMed, Web of Science, and the Cochrane Library, as well as the China National Knowledge Infrastructure (CNKI), Wanfang, and VIP for studies related to chronic fatigue syndrome (CFS) or myalgic encephalomyelitis (ME/CFS) and acupuncture or moxibustion, from database inception to August 2024. The study search terms were adapted to each database and combined subject headings and free-text words, including “chronic fatigue syndrome,” “CFS,” “myalgic encephalomyelitis,” “ME/CFS,” “acupuncture,” “moxibustion,” “electroacupuncture,” and related synonyms. We also screened the reference lists of relevant key articles and existing systematic reviews to identify additional relevant publications.

**Study selection and inclusion criteria**

We included peer-reviewed articles that addressed at least one of the following: (1) epidemiology of CFS/ME, including prevalence, incidence, risk factors, or susceptible populations; (2) diagnostic approaches, including clinical diagnostic criteria, biomarkers, and imaging findings; (3) genetic or pathophysiological mechanisms of CFS, with a focus on immune, neuroendocrine, and metabolic abnormalities; and (4) clinical or mechanistic studies of acupuncture and/or moxibustion for CFS or closely related fatigue syndromes. For sections on clinical efficacy, priority was given to randomized controlled trials (RCTs), prospective clinical studies, and systematic reviews or meta-analyses of acupuncture and moxibustion. Observational studies, narrative reviews, and basic science or animal experimental studies were considered if they provided important information on pathogenesis or potential mechanisms of acupuncture relevant to CFS. Articles were included if they were published in English or Chinese. Conference abstracts, case reports without clear diagnostic information, non-peer-reviewed material, and studies that did not clearly distinguish CFS/ME from other conditions were excluded.

**Data extraction and synthesis**

Two authors independently screened titles and abstracts to identify potentially relevant studies and then reviewed the full text to decide on final inclusion. Disagreements were resolved through discussion or consultation with a third author. For each included study, we extracted basic information (first author, year, country/region), study design, sample size, diagnostic criteria, main interventions (including acupuncture or moxibustion protocols where applicable), control conditions, and key outcomes (e.g., fatigue scales, quality-of-life measures, laboratory or imaging indicators). Given the heterogeneity of study designs, populations, and outcome measures, we performed a qualitative synthesis rather than a formal meta-analysis and organized the narrative according to major themes (epidemiology, diagnosis, genetics, pathophysiology, basic theory of acupuncture and moxibustion, clinical evidence, and future research directions).

**Quality assessment**

Since this work is a narrative review, we did not apply a single formal risk-of-bias tool to all included studies. However, when considering clinical trials and systematic reviews on acupuncture and moxibustion, we considered key methodological features such as randomization procedures, allocation concealment, blinding of participants and outcome assessors, sample size, completeness of follow-up, and clarity of outcome reporting. These judgements were essential for informing our interpretation of the evidence and are reflected in the main text section “Methodological quality and limitations of current evidence,” where we summarize common sources of bias and heterogeneity in the existing literature on acupuncture and moxibustion for CFS.
